# Projections from the posterolateral olfactory amygdala to the ventral striatum: neural basis for reinforcing properties of chemical stimuli

**DOI:** 10.1186/1471-2202-8-103

**Published:** 2007-11-29

**Authors:** Isabel Ubeda-Bañon, Amparo Novejarque, Alicia Mohedano-Moriano, Palma Pro-Sistiaga, Carlos de la Rosa-Prieto, Ricardo Insausti, Fernando Martinez-Garcia, Enrique Lanuza, Alino Martinez-Marcos

**Affiliations:** 1 Laboratorio de Neuroanatomía Humana, Departamento de Ciencias Médicas, Facultad de Medicina, Centro Regional de Investigaciones Biomédicas, Universidad de Castilla-La Mancha, 02006 Albacete, Spain; 2 Laboratory of Comparative & Functional Neuroanatomy, Departament de Biologia Funcional, Facultat de Ciències Biològiques, Universitat de Valencia, 46100 Burjassot, Valencia, Spain; 3 Departament de Biologia Cellular, Facultat de Ciències Biològiques, Universitat de Valencia, 46100 Burjassot, Valencia, Spain

## Abstract

**Background:**

Vertebrates sense chemical stimuli through the olfactory receptor neurons whose axons project to the main olfactory bulb. The main projections of the olfactory bulb are directed to the olfactory cortex and olfactory amygdala (the anterior and posterolateral cortical amygdalae). The posterolateral cortical amygdaloid nucleus mainly projects to other amygdaloid nuclei; other seemingly minor outputs are directed to the ventral striatum, in particular to the olfactory tubercle and the islands of Calleja.

**Results:**

Although the olfactory projections have been previously described in the literature, injection of dextran-amines into the rat main olfactory bulb was performed with the aim of delimiting the olfactory tubercle and posterolateral cortical amygdaloid nucleus in our own material. Injection of dextran-amines into the posterolateral cortical amygdaloid nucleus of rats resulted in anterograde labeling in the ventral striatum, in particular in the core of the nucleus accumbens, and in the medial olfactory tubercle including some islands of Calleja and the cell bridges across the ventral pallidum. Injections of Fluoro-Gold into the ventral striatum were performed to allow retrograde confirmation of these projections.

**Conclusion:**

The present results extend previous descriptions of the posterolateral cortical amygdaloid nucleus efferent projections, which are mainly directed to the core of the nucleus accumbens and the medial olfactory tubercle. Our data indicate that the projection to the core of the nucleus accumbens arises from layer III; the projection to the olfactory tubercle arises from layer II and is much more robust than previously thought. This latter projection is directed to the medial olfactory tubercle including the corresponding islands of Calleja, an area recently described as critical node for the neural circuit of addiction to some stimulant drugs of abuse.

## Background

Vertebrates sense chemical stimuli through the olfactory epithelium, where receptor neurons [1] send axons to the main olfactory bulb [2]. Axons of the projection (mitral) cells of the main olfactory bulb are directed to the olfactory cortex and olfactory amygdala, specifically the anterior and posterolateral cortical amygdaloid nuclei [3-5]. The posterolateral cortical amygdaloid nucleus projects to other amygdaloid nuclei; whereas other seemingly minor outputs are directed to the ventral striatum, in particular to the core of the nucleus accumbens and olfactory tubercle [6-11]. The present data indicate that this projection is not minor in terms of robustness and it is directed to the medial, but not to the lateral, olfactory tubercle and islands of Calleja as well as to some of the cell bridges of the ventral striatum. Furthermore, the projections to the medial olfactory tubercle and to the core of the nucleus accumbens originate in different layers of the posterolateral cortical amygdaloid nucleus. Interestingly, it has recently been demonstrated that the classical reward circuit from the dopaminergic cells of the ventral tegmental area to the nucleus accumbens appears to be irrelevant in mediating the reinforcing properties that male-derived chemicals possess for females [12]. In contrast, the amygdalo-striatal projections might play a critical role in this behavioral response [13,14]. In addition, the medial portion of the olfactory tubercle appears to play a key role in the reinforcing properties of cocaine [15] and amphetamines [16]. Accordingly, the present data are particularly interesting in the context of the current view of the functional and anatomical reward circuits in the ventral striatum [17].

Regarding the nomenclature used in this report, it is necessary to make some comments. According to previous descriptions of efferent projections of the main olfactory bulb, the olfactory amygdala is composed of the anterior cortical amygdaloid nucleus and the posterolateral cortical amygdaloid nucleus, as opposed to the vomeronasal amygdala, which is mainly composed of the medial nucleus and the posteromedial cortical amygdaloid nucleus [7,18-21]. However, the anterior cortical amygdaloid nucleus has been recently demonstrated also to receive inputs from the accessory olfactory bulb and, consequently, should be considered as a mixed chemosensory area [4]. In the present study, therefore, the olfactory amygdala was limited to the posterolateral cortical amygdaloid nucleus. However, the ventral striatal territories included in this study were the nucleus accumbens (which includes the shell, core and rostral pole) [22,23], the olfactory tubercle [10], the cell bridges linking the ventral shell of the accumbens with the olfactory tubercle, and the striatopallidal system of the islands of Calleja [24].

## Results

In the present work, the projections from the olfactory amygdala, and particularly those from the posterolateral cortical amygdaloid nucleus, to the ventral striatum have been analyzed in rats. Olfactory projections have been previously described in studies based on lesion degeneration, autoradiography and horseradish peroxidase [20,25-27]. However, we performed injections of dextran-amines, considered to be among the most sensitive tracers [28], into the main olfactory bulb in order to produce our own material in which to identify olfactory structures in detail [4]. Subsequently, the injections were aimed at the posterolateral cortical amygdaloid nucleus, and the anterograde labeling was analyzed in the ventral striatum. These pathways have been previously traced using autoradiographic methods in rats and cats [8], and in hamsters [7], and using *Phaseolus vulgaris *leucoagglutinin (PHA-L) in rats [29]. Our results show a projection to the ventral striatum that is denser than previously described, probably because dextran-amines are more sensitive anterograde tracers than are tritiated amino-acids [30] and because the injections of PHA-L were located in deep layers of the posterior cortical amygdala [29] and are therefore not comparable to those of the present report (see discussion). Finally, injections of the retrograde tracer Fluoro-Gold were aimed at different sites of the ventral striatum to allow retrograde confirmation of the amygdalo-striatal connections. Throughout the results, the nomenclature of Paxinos and Watson has been used [31].

### Injections in the main olfactory bulb

**Figure 1:**
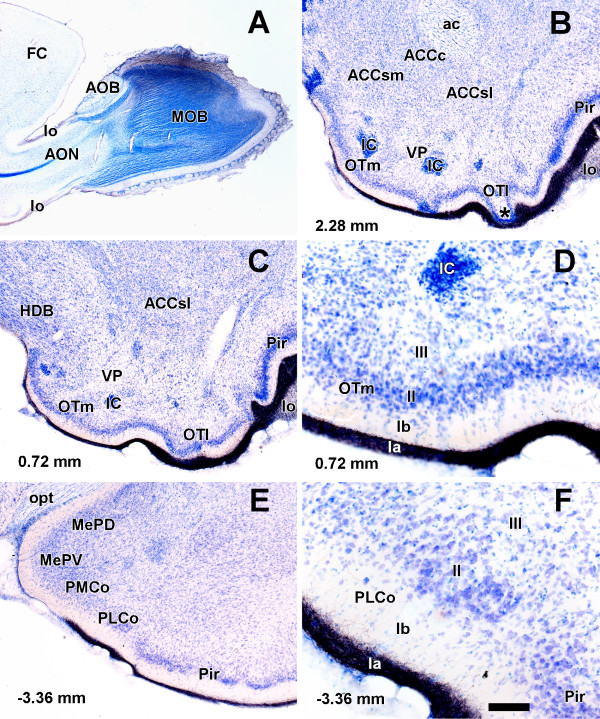
**Projections from the main olfactory bulb**. Parasaggital (A) and coronal (B-F) Nissl-counterstained sections of the rat brain showing anterograde labeling (B-F) after a biotinylated dextran-amine injection into the main olfactory bulb (A). For abbreviations, see list. Calibration bar: A 1250 μm; B, C, E 400 μm; D, F 100 μm.

Six injections of biotinylated dextran-amine were placed at the main olfactory bulb. Case 7605 is described as a representative case. The results of these injections were in agreement with previous descriptions of the projections from the main olfactory bulb [3-5,20,21,26]. The description of the results is focused on our areas of interest, i.e., the olfactory tubercle and the posterolateral cortical amygdaloid nucleus. The injection affected the dorsal portion of the mitral cell layer of the main olfactory bulb (Fig. 1A). The accessory olfactory bulb was not contaminated by the injection site. Labeled fibers coursing dorsally in the lateral olfactory tract traveled between the mitral and granule cell layers of the accessory olfactory bulb [4] and ventrally below the anterior olfactory nucleus (Fig. 1A). In the rostral (Fig. 1B) and caudal (Fig. 1C) levels of the olfactory tubercle, labeled fibers were found in layer I, sublamina Ia [32] (Fig. 1D), throughout its medio-lateral and antero-posterior axes. Interestingly, in some parts of the lateral olfactory tubercle, sublamina Ib seemed to disappear and labeled fibers were seen to be adjacent to the cell bodies of layer II (asterisk in 1B). The bundle of labeled fibers became progressively thinner from lateral to medial (Fig. 1B,C). In fact, in the more medial portion, sublamina Ib also narrowed and cell bodies bordered the labeled fibers in sublamina Ia. Caudally, labeled fibers occupied the most superficial portion of layer I in the posterolateral cortical amygdaloid nucleus, which could be easily recognized on cytoarchitectonic grounds, as well as in the piriform cortex (Fig. 1E,F). Sublamina Ia appeared thicker in the posterolateral cortical amygdaloid nucleus compared with the piriform cortex (Fig. 1E).

### Injections in the posterolateral cortical amygdaloid nucleus

**Figure 2:**
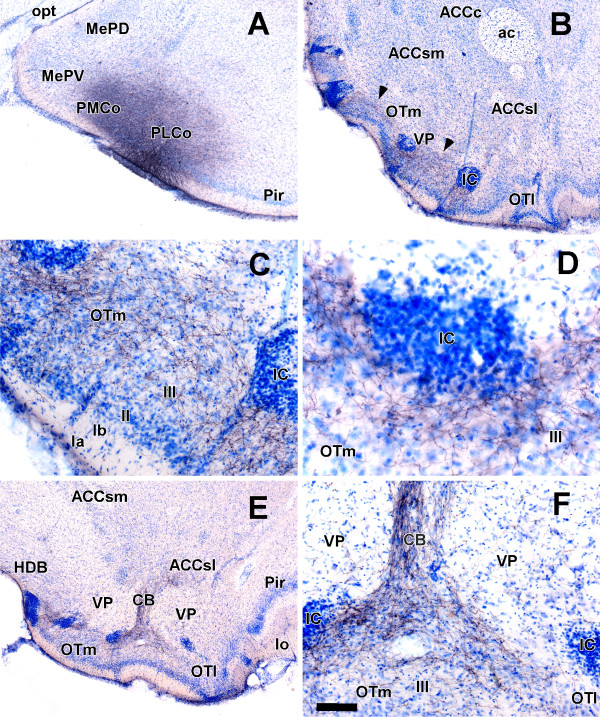
**Projections from the posterolateral cortical amygdaloid nucleus**. Coronal Nissl-counterstained sections of the rat brain showing anterograde labeling (B-F) after a biotinylated dextran-amine injection into the posterolateral cortical amygdaloid nucleus (A). For abbreviations, see list. Calibration bar: A, B, E 400 μm; C, F 100 μm; D 50 μm.

Four injections of biotinylated dextran-amine were placed in the posterolateral cortical amygdaloid nucleus. Case 7705 is described as an example. The injection site affected a few cells of layer II (Fig. 2A). The description of the labeling is limited to the ventral striatum. Rostrally, labeled fibers were distributed along the medial olfactory tubercle, particularly in layers II and III and surrounding the medial islands of Calleja (Fig. 2B–D). More caudally, the pattern of labeling was similar in the medial olfactory tubercle and islands of calleja (Fig. 2E,F). The labeled terminal fields in the olfactory tubercle/islands of Calleja on the one hand, and in the lateral shell of the nucleus accumbens on the other were linked by dense labeling in some of the intervening cell bridges of the ventral striatum (Fig. 2E,F). Some fibers also could be observed in the caudal lateral portion of the shell of the nucleus accumbens (Fig. 2E) and the ventral pallidum (Fig. 2F).

**Figure 3:**
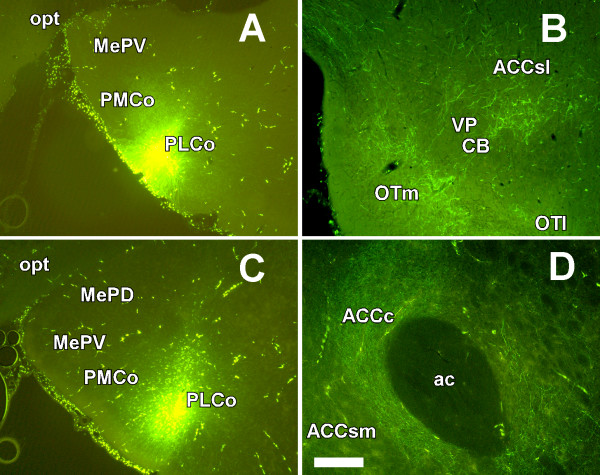
**Projections from layers of the posterolateral cortical amygdaloid nucleus**. Coronal sections of rat brain showing anterograde labeling (B, D) after injection of fluorescein dextran-amine into layers II (A) and III (C) of the posterolateral cortical amygdaloid nucleus, respectively. For abbreviations, see list. Calibration bar: A, C 400 μm; B, D 200 μm.

Four injections of fluorescein-labeled dextran-amine were targeted to the posterolateral cortical amygdaloid nucleus. Case 8705 received an injection similar to that of case 7705, which mostly affected layer II (Fig. 3A). As expected, the pattern of labeling was similar to that obtained after biotinylated dextran-amine injections, including the medial olfactory tubercle, island of Calleja, cell bridges of the ventral striatum and the caudal ventrolateral portion of the shell of the nucleus accumbens. A few labeled fibers were also observed in the ventral pallidum (Fig. 3B). Injection of case 9905 involved layer III of the posterolateral cortical amygdaloid nucleus (Fig. 3C). The pattern of labeling in the ventral striatum was similar to the one described for the previous injections, but dense anterograde labeling was observed in the core of nucleus accumbens (Fig. 3D; compare with Fig. 2B).

### Injections in the ventral striatum

**Figure 4:**
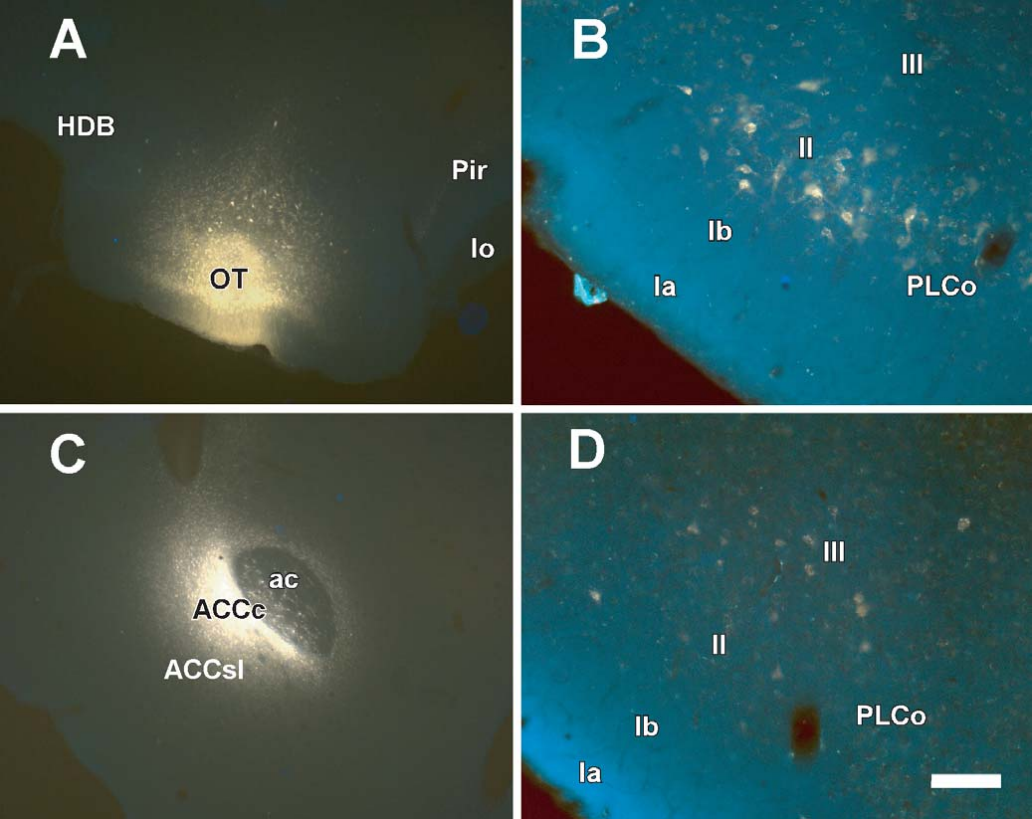
**Projections to the ventral striatum**. Coronal sections of rat brain showing retrograde labeling (B, D) after Fluoro-Gold injections into the olfactory tubercle (A) and core of the nucleus accumbens (C), respectively. For abbreviations, see list. Calibration bar: A, C 400 μm; B, D 100 μm.

Six injections of Fluoro-Gold affected the olfactory tubercle. The injection in case 1207 involved layers II and III of the caudal olfactory tubercle (Fig. 4A). Apart from retrograde-labeled cells in areas such as the basolateral amygdaloid complex and the amygdalo-hippocampal transition area (not shown), a number of labeled cells were observed in layer II of the posterolateral cortical amygdaloid nucleus (Fig. 4B).

Five injections of Fluoro-Gold were placed at the core of the nucleus accumbens. Case 7307 is described as an example. The injection site was restricted to the core of the nucleus (Fig. 4C). Retrograde labeling was observed, in addition to the other structures (see [11]), in layer III of the posterolateral cortical amygdaloid nucleus (Fig. 4D).

## Discussion

The aim of the present work was to characterize the direct olfactory inputs from olfactory-recipient amygdaloid structures to ventral striatal territories that may underlie the reinforcing properties of chemical stimuli. A direct projection from the posterolateral cortical amygdaloid nucleus to the medial olfactory tubercle, islands of Calleja and the ventrolateral shell and core of the nucleus accumbens has been described. Injections of anterograde tracers into layer II of the posterolateral cortical amygdaloid nucleus (cases 7705 and 8705) resulted in anterograde labeling in the medial olfactory tubercle and islands of Calleja and sparse labeling in the ventrolateral shell of the nucleus accumbens and ventral pallidum. Retrograde confirmation was obtained by injection into the olfactory tubercle (case 1207). Injections of anterograde tracers involving layer III (case 9905) gave rise, in addition, to conspicuous anterograde labeling in the core of the nucleus accumbens. Retrograde confirmation of this projection was obtained following injection into the core of the nucleus accumbens (case 7307). Therefore, layers II and III of the posterolateral cortical amygdaloid nucleus send differential projections to the medial olfactory tubercle and islands of Calleja, and to the core of the nucleus accumbens, respectively.

### Neuroanatomical remarks

The efferent projections from the main olfactory bulb have been described to reach a number of structures in the basal telencephalon, including the anterior olfactory nucleus, the olfactory tubercle, the piriform cortex, the entorhinal cortex, and the anterior and posterolateral cortical amygdalae [4,5,21,25,26,33,34]. Our results confirm these descriptions. In contrast to the majority of previous studies, however, we used biotinylated dextran-amine, considered to be among the most sensitive anterograde tracers [30]. This has allowed comparison of the thickness of sublamina Ia of layer I [32,33] among the olfactory structures.

The efferent connections of the posterolateral cortical amygdaloid nucleus have been studied previously in different species [7,8,29]. In rats, injections into the periamygdaloid cortex, i.e., the posterolateral cortical amygdaloid nucleus in Scalia and Winans' nomenclature [20], resulted in anterograde labeling mostly concentrated in layer II of the olfactory tubercle and around medially located islands of Calleja. No labeling was observed in the nucleus accumbens or ventral putamen [8]. In hamsters, efferents from the posterolateral cortical amygdaloid nucleus terminate in layers II and III of the olfactory tubercle, mostly in the medial part. Labeling was also observed around, but not whithin the islands of Calleja [7]. More recently, and also in rats, on the basis of injections in the deepest part of the posterolateral cortical amygdaloid nucleus, this nucleus has been reported to "provide a very sparse input to the fundus of the striatum and to medial parts of the olfactory tubercle", with fibers also reaching the core of the nucleus accumbens [29]. Our present results confirm this findings and expand previous descriptions because some of the cited reports used the autoradiographic method of tritiated amino acids [7,8], which is less sensitive and produces large injection sites compared with iontophoretic injections of more modern tracers. Our data provide a more accurate account of these projections. Conversely, iontophoretic injections of *Phaseolus vulgaris *leucoagglutinin (PHA-L), which is as sensitive as biotinylated dextran-amine [30], were placed in the deepest part of the posterolateral cortical amygdaloid nucleus [29] (a location that is usually considered to be within the amygdalo-hippocampal area rather than the posterolateral cortical nucleus) and the findings were, therefore, only partially comparable to the present results. Our results demonstrate in detail that the input from the posterolateral cortical amygdala extends in layer II and layer III of the olfactory tubercle and that varicose fibers not only surround but enter the islands of Calleja (Fig. 2D). Furthermore, we have confirmed this amygdalo-striatal projection by using retrograde tracing. Although previous studies already suggested that the olfactory tubercle, islands of Calleja and nucleus accumbens were targeted by projections from the cortical amygdala [10,35-37], our results demonstrate that, at least in the posterolateral cortical amygdala, the inputs to the olfactory tubercle and islands of Calleja mostly arise from layer II, whereas those directed to the nucleus accumbens core arise from layer III.

### Functional considerations

The olfactory tubercle receives direct olfactory inputs through layer I from the main olfactory bulb (Fig. 1), and indirect olfactory inputs in layers II and III from the posterolateral cortical amygdaloid nucleus (Figs. 2, 3, 4). The function of this indirect projection is unknown. In addition, the cells of the islands of Calleja [38] could receive indirect olfactory inputs from the posterolateral cortical amygdaloid nucleus (Fig. 2), the functional implications of which are also unknown.

A number of findings suggest a possible role for such projections. Amygdalo-striatal pathways, particularly the projections from the basolateral amygdala to the nucleus accumbens, have been implicated in addiction and reward phenomena [39-41]. Recently, the involvement of the ventral striatum in reward has been redefined to include not only the nucleus accumbens, but also the olfactory tubercle, particularly its medial portion [15,16]. The reward circuits in the ventral striatum would conform two functionally distinct subsystems, a medial one composed of the medial shell of the nucleus accumbens and the medial olfactory tubercle and a lateral subsystem that would include the core and lateral shell of nucleus accumbens plus the lateral aspect of the olfactory tubercle [17]. Conversely, it has been demonstrated that some chemical signals, such as sexual pheromones, are innately attractive, i.e., intrinsically reinforcing [42,43]. Furthermore, odorants associated with involatile pheromones have been suggested to become reinforcing through an olfactory-vomeronasal associative learning process that may involve the basolateral amygdala [44]. The posteromedial cortical amygdaloid nucleus (part of the vomeronasal amygdala) also projects to the olfactory tubercle, islands of Calleja, cell bridges of the ventral striatum and shell of the nucleus accumbens [14], which suggests that the association of olfactory and vomeronasal information in rewarding events could also take place in the ventral striatum. Contrary to the traditional view of separate functional and anatomical axes through the forebrain for the olfactory and vomeronasal systems [20,45], recent data indicate that secondary olfactory and vomeronasal projections converge in the rostral basal telencephalon [4]. Similarly, olfactory and vomeronasal information converges in the posteromedial cortical amygdaloid nucleus [46]. New data from our group indicate that vomeronasal and olfactory information could converge in the ventral striatum through the posterolateral (present report) and posteromedial [14] cortical amygdalae, respectively.

## Conclusion

The present study demonstrates an indirect olfactory input from the posterolateral cortical amygdaloid nucleus to the ventral striatum. Neurons of layer II of the posterolateral cortical amygdaloid nucleus project to layers II and III of the olfactory tubercle and islands of Calleja; in contrast, cells in layer III project to the core of the nucleus accumbens. These projections could constitute the neural basis for processing the reinforcing properties of olfactory stimuli.

## Methods

### Animals and ethical considerations

Twenty-five adult male and female Sprague-Dawley rats from the University Hospital of Albacete were used in the present study. Experimental procedures were carried out according to the guidelines of the European Community on welfare of research animals (directive 86/609/EEC) and were approved by the Ethical Committees of Animal Research of the University of Castilla-La Mancha for grants PAC-05-007 and PCC08-0064.

### Tracer injections

Twenty-five rats were injected intraperitoneally with a combined dose of ketamine hydrochloride (Ketolar^®^, Parke-Davis, Madrid, 1.5 ml/kg, 75 mg/kg) and xylazine (Xilagesic^®^, Calier, Barcelona, 0.5 ml/kg, 10 mg/kg). The animals were placed into a stereotaxic apparatus and the skull was trepanned at the intended injection site. Iontophoretic injections of dextran-amines conjugated to biotin (BDA) or fluorescein (FDA) (10% in phosphate buffered saline, 10,000 MW, lysine fixable, Molecular Probes, Eugene, OR) and Fluoro-Gold^® ^(FG) (2% in saline solution, methanesulfonate hydroxystilbamidine, Biotium, Hayward, CA) were performed. Tracers were delivered from micropipettes (10–50 μm diameter tips) by means of positive current pulses (7/7 sec, 2–7 μA, 10–20 min).

### Perfusion, cutting and tracer detection

Five to eight days later, animals were anesthetized (as described above) and transcardially perfused with saline solution followed by 4% paraformaldehyde in phosphate buffer. The brains were postfixed for 4 hours and cryoprotected overnight with 30% buffered sucrose. Series of frontal sections (50 μm) were obtained with a freezing microtome. For BDA detection, endogenous peroxidase activity was quenched using 1% H_2_O_2 _(in 0.05 M Tris buffered saline, pH 7.6, for 30 min). Sections were incubated for 2 hours in avidin-biotin complex (ABC elite kit, Vector, Burlingame, CA; diluted in 0.05 M Tris buffered saline, pH 7.6) and visualized with 0.025% 3,3'-diaminobenzidine (Sigma, St. Louis, MO) diluted in 0.05 M Tris buffer (pH 8.0) with 0.1% ammonium nickel sulfate and 0.01% H_2_O_2_. Sections were Nissl-counterstained, mounted, dried and coverslipped (see [4,19], for further details of procedures).

### Methodological considerations

Assuming that tract-tracing techniques are largely based on qualitative descriptions, some methodological considerations are necessary when comparing the previous literature on the amygdalo-striatal projections with the present results regarding the relative density of projections. Relatively large injections of tritiated amino-acids into the posterolateral cortical amygdaloid nucleus gave rise to a moderate labeling of grains in the olfactory tubercle and islands of Calleja [7,8]. As commented on in the results and discussion, other studies using *Phaseolus vulgaris *leucoagglutinin [29] are not comparable to the present report because the injections are placed in deep layers of the amygdala. In the present report, fully restricted injections of dextran-amines into the posterolateral cortical amygdaloid nucleus (Figs. 2A, 3A,C) gave rise to a dense meshwork of labeled fibers in the cell bridges and medial olfactory tubercle (Fig. 2B–F, 3B). Therefore, the density of this amygdalo-striatal projection has been previously underestimated, probably due to technical limitations.

## Abbreviations

ac anterior commissure

ACCc nucleus accumbens, core

ACCsl nucleus accumbens, shell lateral

ACCsm nucleus accumbens, shell medial

AOB accessory olfactory bulb

AON anterior olfactory nucleus

BDA biotinylated-labeled dextran-amine

CB cell bridges of the ventral striatum

FDA fluorescein-labeled dextran-amine

FC frontal cortex

FG Fluoro Gold

HDB nucleus of the horizontal limb of the diagonal band

IC islands of Calleja

lo lateral olfactory tract

MePD medial amygdaloid nucleus, posterodorsal

MePV medial amygdaloid nucleus, posteroventral

MOB main olfactory bulb

opt optic tract

OT olfactory tubercle

OTl lateral olfactory tubercle

OTm medial olfactory tubercle

Pir piriform cortex

PLCo posterolateral cortical amygdaloid nucleus

PMCo posteromedial cortical amygdaloid nucleus

VP ventral pallidum

Ia layer I, sublamina Ia

Ib layer I, sublamina Ib

II layer II

III layer III

## Authors' contributions

IUB, AN, AMM, PPS, and CRP participated in the design and performance of experiments and participated in the preparation of text and figures of the manuscript draft. RI, FMG, EL and AMM^§ ^conceived the study, including the analysis of results, and wrote the manuscript. AMM^§ ^coordinated the study. All authors read and approved the final manuscript.

## References

[B1] Buck LB (1996). Information coding in the vertebrate olfactory system. Annu Rev Neurosci.

[B2] Mori K, Nagao H, Yoshihara Y (1999). The olfactory bulb: coding and processing of odor molecule information. Science.

[B3] Martinez-Marcos A, Halpern M (2006). Efferent connections of the main olfactory bulb in the opossum (Monodelphis domestica): a characterization of the olfactory entorhinal cortex in a marsupial. Neurosci Lett.

[B4] Pro-Sistiaga P, Mohedano-Moriano A, Ubeda-Banon I, Mar Arroyo-Jimenez M, Marcos P, Artacho-Perula E, Crespo C, Insausti R, Martinez-Marcos A (2007). Convergence of olfactory and vomeronasal projections in the rat basal telencephalon. J Comp Neurol.

[B5] Shipley MT, Ennis M, Puche AC, Paxinos G (2004). Olfactory system. The Rat Nervous System.

[B6] Chiba T (2000). Collateral projection from the amygdalo--hippocampal transition area and CA1 to the hypothalamus and medial prefrontal cortex in the rat. Neurosci Res.

[B7] Kevetter GA, Winans SS (1981). Connections of the corticomedial amygdala in the golden hamster. II. Efferents of the "olfactory amygdala". J Comp Neurol.

[B8] Krettek JE, Price JL (1978). Amygdaloid projections to subcortical structures within the basal forebrain and brainstem in the rat and cat. J Comp Neurol.

[B9] Price JL, Slotnick BM, Revial MF (1991). Olfactory projections to the hypothalamus. J Comp Neurol.

[B10] Newman R, Winans SS (1980). An experimental study of the ventral striatum of the golden hamster. II. Neuronal connections of the olfactory tubercle. J Comp Neurol.

[B11] Brog JS, Salyapongse A, Deutch AY, Zahm DS (1993). The patterns of afferent innervation of the core and shell in the "accumbens" part of the rat ventral striatum: immunohistochemical detection of retrogradely transported fluoro-gold. J Comp Neurol.

[B12] Martinez-Hernandez J, Lanuza E, Martinez-Garcia F (2006). Selective dopaminergic lesions of the ventral tegmental area impair preference for sucrose but not for male sexual pheromones in female mice. Eur J Neurosci.

[B13] Lanuza E, Novejarque A, Martinez-Ricos J, Martinez-Hernandez J, Agustin-Pavon C, Martinez-Garcia F (2007). Sexual pheromones and the evolution of the reward system of the brain: The chemosensory function of the amygdala. Brain Res Bull.

[B14] Ubeda-Banon I, Novejarque A, Mohedano-Moriano A, Pro-Sistiaga P, Insausti R, Martinez-Garcia F, Lanuza E, Martinez-Marcos A (2007). Vomeronasal inputs to the rodent ventral striatum. Brain Res Bull.

[B15] Ikemoto S (2003). Involvement of the olfactory tubercle in cocaine reward: intracranial self-administration studies. J Neurosci.

[B16] Ikemoto S, Qin M, Liu ZH (2005). The functional divide for primary reinforcement of D-amphetamine lies between the medial and lateral ventral striatum: is the division of the accumbens core, shell, and olfactory tubercle valid?. J Neurosci.

[B17] Ikemoto S (2007). Dopamine reward circuitry: Two projection systems from the ventral midbrain to the nucleus accumbens-olfactory tubercle complex. Brain Res Rev.

[B18] Kevetter GA, Winans SS (1981). Connections of the corticomedial amygdala in the golden hamster. I. Efferents of the "vomeronasal amygdala". J Comp Neurol.

[B19] Mohedano-Moriano A, Pro-Sistiaga P, Ubeda-Banon I, Crespo C, Insausti R, Martinez-Marcos A (2007). Segregated pathways to the vomeronasal amygdala: differential projections from the anterior and posterior divisions of the accessory olfactory bulb. Eur J Neurosci.

[B20] Scalia F, Winans SS (1975). The differential projections of the olfactory bulb and accessory olfactory bulb in mammals. J Comp Neurol.

[B21] Skeen LC, Hall WC (1977). Efferent projections of the main and the accessory olfactory bulb in the tree shrew (Tupaia glis). J Comp Neurol.

[B22] Zahm DS, Brog JS (1992). On the significance of subterritories in the "accumbens" part of the rat ventral striatum. Neuroscience.

[B23] Newman R, Winans SS (1980). An experimental study of the ventral striatum of the golden hamster. I. Neuronal connections of the nucleus accumbens. J Comp Neurol.

[B24] Fallon JH, Loughlin SE, Ribak CE (1983). The islands of Calleja complex of rat basal forebrain. III. Histochemical evidence for a striatopallidal system. J Comp Neurol.

[B25] Davis BJ, Macrides F, Youngs WM, Schneider SP, Rosene DL (1978). Efferents and centrifugal afferents of the main and accessory olfactory bulbs in the hamster. Brain Res Bull.

[B26] Devor M (1976). Fiber trajectories of olfactory bulb efferents in the hamster. J Comp Neurol.

[B27] Kosel KC, Van Hoesen GW, West JR (1981). Olfactory bulb projections to the parahippocampal area of the rat. J Comp Neurol.

[B28] Reiner A, Veenman CL, Medina L, Jiao Y, Del Mar N, Honig MG (2000). Pathway tracing using biotinylated dextran amines. J Neurosci Methods.

[B29] Canteras NS, Simerly RB, Swanson LW (1992). Connections of the posterior nucleus of the amygdala. J Comp Neurol.

[B30] Kobbert C, Apps R, Bechmann I, Lanciego JL, Mey J, Thanos S (2000). Current concepts in neuroanatomical tracing. Prog Neurobiol.

[B31] Paxinos G, Watson C (2005). The Rat Brain in Stereotaxic Coordinates.

[B32] Price JL (1973). An autoradiographic study of complementary laminar patterns of termination of afferent fibers to the olfactory cortex. J Comp Neurol.

[B33] Price JL, Finger TT, Silver WL (1987). The central olfactory and accessory olfactory systems. Neurobiology of Taste and Smell.

[B34] Shammah-Lagnado SJ, Negrao N (1981). Efferent connections of the olfactory bulb in the opossum (Didelphis marsupialis aurita): a Fink-Heimer study. J Comp Neurol.

[B35] Fallon JH, Riley JN, Sipe JC, Moore RY (1978). The islands of Calleja: organization and connections. J Comp Neurol.

[B36] Fallon JH (1983). The islands of Calleja complex of rat basal forebrain II: connections of medium and large sized cells. Brain Res Bull.

[B37] Phillipson OT, Griffiths AC (1985). The topographic order of inputs to nucleus accumbens in the rat. Neuroscience.

[B38] Meyer G, Gonzalez-Hernandez T, Carrillo-Padilla F, Ferres-Torres R (1989). Aggregations of granule cells in the basal forebrain (islands of Calleja): Golgi and cytoarchitectonic study in different mammals, including man. J Comp Neurol.

[B39] Cador M, Robbins TW, Everitt BJ (1989). Involvement of the amygdala in stimulus-reward associations: interaction with the ventral striatum. Neuroscience.

[B40] Everitt BJ, Morris KA, O'Brien A, Robbins TW (1991). The basolateral amygdala-ventral striatal system and conditioned place preference: further evidence of limbic-striatal interactions underlying reward-related processes. Neuroscience.

[B41] Everitt BJ, Parkinson JA, Olmstead MC, Arroyo M, Robledo P, Robbins TW (1999). Associative processes in addiction and reward. The role of amygdala-ventral striatal subsystems. Ann N Y Acad Sci.

[B42] Moncho-Bogani J, Lanuza E, Hernandez A, Novejarque A, Martinez-Garcia F (2002). Attractive properties of sexual pheromones in mice: innate or learned?. Physiol Behav.

[B43] Martinez-Ricos J, Agustin-Pavon C, Lanuza E, Martinez-Garcia F (2007). Intraspecific communication through chemical signals in female mice: reinforcing properties of involatile male sexual pheromones. Chem Senses.

[B44] Moncho-Bogani J, Martinez-Garcia F, Novejarque A, Lanuza E (2005). Attraction to sexual pheromones and associated odorants in female mice involves activation of the reward system and basolateral amygdala. Eur J Neurosci.

[B45] Halpern M, Martinez-Marcos A (2003). Structure and function of the vomeronasal system: an update. Prog Neurobiol.

[B46] Licht G, Meredith M (1987). Convergence of main and accessory olfactory pathways onto single neurons in the hamster amygdala. Exp Brain Res.

